# Environmental Assessment Tools for Shared Residential Settings for Individuals Living With Dementia

**DOI:** 10.1093/geroni/igab046.1013

**Published:** 2021-12-17

**Authors:** Margaret Calkins, Migette Kaup, Addie Abushousheh

**Affiliations:** 1 IDEAS Institute, Cleveland Hts, Ohio, United States; 2 Kansas State University, Manhattan, Kansas, United States; 3 Center for Health Design, West Bend, Wisconsin, United States

## Abstract

The overarching goal of conducting research in and on settings for individuals living with dementia is to be able to identify associations between features, or set(s) of features, and outcomes of interest. A major challenge to this goal is the disconnect between the rapidly changing arena of LTC design over the past 20 years toward more residential-styled settings and existing assessment tools, many of which were developed over 20 years ago, and which are based on and reflect more traditional models of care, service delivery and design. This session examines the strengths and limitations of both existing and emerging assessment tools from the US, Canada, UK and Australia. More recently developed tools are more directly focused on the values of person-centered care than older tools.

